# Real‐World Longitudinal Data on the Impact of Hydroxychloroquine Blood Level Monitoring on Lupus Outcomes: Results of a Prospective Longitudinal Cohort Study

**DOI:** 10.1002/acr.80059

**Published:** 2026-05-03

**Authors:** Jay J. Patel, Fauzia Hollangel, Caroline Packee, Amita Kapoor, Brad Rovin, Nathalie Costedoat‐Chalumeau, Shivani Garg

**Affiliations:** ^1^ University of Wisconsin School of Medicine and Public Health Madison; ^2^ Wisconsin National Primate Research Center, University of Wisconsin‐Madison; ^3^ Ohio State University, Wexner Medical Center Columbus; ^4^ National Referral Centre for Rare Autoimmune and Systemic Diseases, Hôpital Cochin, AP‐HP Centre and Université Paris Cité and Centre de Recherche en Epidémiologie et Statistiques (CRESS), Unité Inserm 1153, Université de Paris Cité Paris France; ^5^ Yale School of Medicine New Haven Connecticut

## Abstract

**Objective:**

Hydroxychloroquine (HCQ) is a cornerstone therapy in systemic lupus erythematosus (SLE), but the weight‐based dosing does not account for clinical factors that can introduce individual variability in drug metabolism and clearance. We leveraged longitudinal data from a prospective SLE cohort to identify clinical factors that predict significant variations in HCQ blood levels despite weight‐based HCQ dosing, and we examined if maintaining therapeutic HCQ levels (750–1,150 ng/mL) improved SLE outcomes.

**Methods:**

We analyzed 962 visits from 247 patients in a prospective SLE cohort. Generalized linear mixed models with random intercepts and slopes assessed associations among variables such as chronic kidney disease, HCQ dose, and HCQ blood levels over time. Next, within‐ and between‐subject decomposition models evaluated the impact of intra‐ and inter‐individual changes in HCQ blood levels and therapeutic HCQ blood levels on active SLE and flares over time.

**Results:**

At baseline visit, the mean patient age was 47 years; 91% were female, 66% were White, 45% had subtherapeutic HCQ levels (<750 ng/mL), and 79% received an HCQ dose of ≤5 mg/kg/day. Despite receiving 5 mg/kg/day, patients with kidney function ≤75 mL/min/1.73m^2^ had supratherapeutic levels over time. Next, very low (<200 ng/mL) and subtherapeutic levels (200 to <750 ng/mL) predicted 6.7‐fold and 2.6‐fold higher odds of active SLE vs therapeutic levels over time. At the individual patient level, increases of 100 ng/mL in HCQ levels at each visit predicted 33% and 22% lower odds of active SLE and flares, respectively.

**Conclusion:**

HCQ blood level monitoring could improve SLE control, particularly in patients with estimated glomerular filtration rate ≤75, and enable optimal HCQ use.

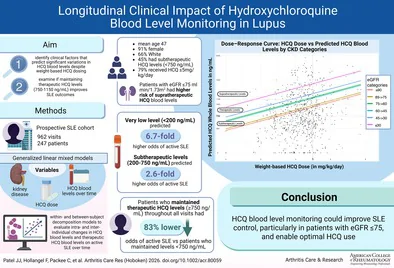

## INTRODUCTION

Hydroxychloroquine (HCQ) is the foundational therapy in the systemic lupus erythematosus (SLE or lupus) therapeutic armamentarium because it prolongs disease‐free and damage‐free survival in patients.^1^ Current guidelines support weight‐based HCQ dosing to minimize eye toxicity without specific recommendations on how to adjust dose based on kidney function or other clinical factors. HCQ is primarily metabolized by the liver, and 40% to 60% of HCQ is excreted by kidneys. However, previous studies lack longitudinal data quantifying the extent to which kidney impairment alters HCQ levels—data that are important to determine safety and effective HCQ dosing beyond current dosing informed only by body weight. Likewise, similar data are lacking on how HCQ blood levels using weight‐based dosing vary in patients with chronic transaminitis or chronic liver disease, cytochrome P450 (CYP) enzymes inhibitor use, and gastric disease.^2^, ^3^, ^4^ Without clear guidance on adjusting HCQ doses in patients with chronic kidney disease (CKD) or chronic transaminitis, doses are either not adjusted or arbitrarily reduced, which could potentially accelerate toxicity risk or increase the risk of SLE flares. Moreover, the “one‐size‐fits‐all” dosing of HCQ exacerbates patients’ worries about irreversible eye toxicity resulting in early discontinuation of the medicine or nonadherence, risking lupus flares and hospitalizations.^5^ Therefore, there is a need for a solution that can bypass clinical variables, nonadherence, and give a better understanding of HCQ dose absorption, particularly in patients with SLE with chronic diseases, to guide personalized HCQ dose based on an individual patient's circumstances.

**SIGNIFICANCE & INNOVATIONS**

- This study identifies that beyond chronic kidney disease Stage 3 and above, patients with an estimated glomerular filtration rate ≤75 mL/min/1.73m^2^ are at higher risk of having supratherapeutic hydroxychloroquine (HCQ) blood levels, risking toxicity in the future. Additionally, female and Asian patients had significantly lower HCQ blood levels despite weight‐based dosing, indicating a potential difference in cytochrome P450 metabolism. Therefore, close HCQ blood level monitoring could improve systemic lupus erythematosus (SLE) control in patients in such populations.
- Having very low (<200 ng/mL) and subtherapeutic (200 to <750 ng/mL) HCQ blood levels can predict 6.7‐fold and 2.6‐fold higher odds of active SLE compared with having therapeutic HCQ blood levels (750 to <1,150 ng/mL) over time.
- At the individual patient level, increases of 100 ng/mL in HCQ levels at each visit predicted 33% and 22% lower odds of active SLE and flares, respectively. Moreover, patients who maintained therapeutic HCQ levels (≥750 ng/mL) throughout all visits had 83% lower odds of active SLE over time compared with patients who maintained subtherapeutic levels (<750 ng/mL).
- Finally, HCQ blood level monitoring was uniformly covered by different payers without significant co‐pay concerns. This study provides essential real‐world evidence that underscores the clinical significance of routine HCQ blood level monitoring to guide optimal HCQ use in clinics.

Therapeutic HCQ blood level monitoring could be the solution because it bypasses clinical variables affecting HCQ absorption or clearance and can guide optimal HCQ dosing at an individual SLE patient level, particularly in those with CKD or chronic transaminitis.^6^, ^7^, ^8^, ^9^, ^10^, ^11^ Global studies, including a global meta‐analysis, have established the clinical significance of using HCQ blood levels as an objective measure to monitor severe nonadherence and also clinical efficacy (lower risk of active lupus or flares).^6^, ^7^, ^8^, ^9^, ^10^, ^11^, ^12^, ^13^, ^14^ Studies on different cohorts supported the use of 750 to 1,150 ng/mL as the target range for HCQ blood level monitoring.^6^, ^9^, ^12^, ^13^, ^14^, ^15^, ^16^, ^17^, ^18^ However, most findings were based on cross‐sectional data. In part because of the absence of longitudinal studies that monitor the clinical effectiveness of HCQ blood level monitoring and guide optimal HCQ dosing, the adoption of HCQ blood levels in clinics has been suboptimal in many parts of the world, particularly the United States. Additional data on how to adjust HCQ dose per HCQ blood levels in patients with and without CKD are needed. Moreover, given that patients with lupus are young and attaining CKD stage ≥3 (estimated glomerular filtration rate [eGFR] <60 mL/min/1.73m^2^) could take time, it is important to know if renal adjustments for HCQ should be considered for other CKD or eGFR thresholds. Finally, longitudinal data showing that HCQ blood levels within the therapeutic range is associated with lower risk of active SLE will be vital to enhance clinical uptake and guide clinicians with precise HCQ dosing based on individual patient risk factors, such as CKD and chronic transaminitis.

Therefore, we leveraged longitudinal data from a prospective SLE cohort to predict HCQ blood levels for weight‐based HCQ dosing stratified by CKD stages, chronic transaminitis categories, and gastric disease. Next, we measured associations between therapeutic HCQ blood levels (750–1,150 ng/mL) and SLE response over time adjusting for individual patient factors.

## METHODS

### Cohort

We abstracted data from a prospective longitudinal SLE cohort (approved by the institutional review board of the University of Wisconsin, Madison, #2019‐0942). Eligible patients were recruited from the University of Wisconsin's Collaborative SLE clinics. Eligibility criteria for the SLE cohort included patients who were aged at least 18 years, had a validated SLE diagnosis,^19^ were on HCQ for at least three months before enrollment, and had been seen in rheumatology clinics at least one to two times a year. Patients were observed over time since the first visit, and random HCQ blood levels and SLE Disease Activity Index 2000 (SLEDAI‐2K) were completed at each follow‐up visit.^20^ Follow‐up visit frequency varied based on routine clinical need showing real‐world practices rather than per protocol follow‐up. Given that the aim of this study was to infer real‐world impact of HCQ blood level monitoring, a requirement for stable background immunosuppressive therapy before enrollment was imposed. All HCQ blood levels were performed at least six hours after the last HCQ dose.^10^, ^21^, ^22^ Therefore, HCQ blood levels in this study were random (non‐trough) measurements that were obtained at least six hours after the last dose, bypassing the peak HCQ levels often seen after the first four to six hours of the last dose.^10^, ^21^, ^22^

### Data

We abstracted key clinical and treatment data from the electronic health records (EHR) of the patients in the SLE cohort. Demographics including age, racial or ethnic group, sex, insurance status, and other variables were abstracted at each visit. Clinical variables, including body weight, active gastric diseases on the problem list (eg, gastroesophageal reflux disease, inflammatory bowel disease, irritable bowel syndrome, peptic ulcer disease, gastritis) or a gastric procedure (eg, gastric bypass), eGFR per the 2021 CKD–Epidemiology Collaboration equation,^23^ and liver function tests (aspartate aminotransferase [AST] and alanine aminotransferase [ALT]) were abstracted from the EHR. Medication data including HCQ dose, immunosuppressants, prednisone (or other equivalent steroid) dose, CYP inhibitors (Supplementary File 1), and chronic use of gastric medicines (eg, pantoprazole, omeprazole, famotidine) at each visit were abstracted. Medication adherence was assessed using three‐month proportion of days covered (PDC) derived from pharmacy refill data in the EHR. Additionally, SLEDAI‐2K scores, Systemic Lupus International Collaborating Clinics Damage Index (SDI),^24^ and abnormal eye examination reporting HCQ toxicity were abstracted at each visit.

Finally, HCQ levels from each visit were abstracted. HCQ levels were measured using a validated high‐performance liquid chromatography mass spectrometry assay.^6^ The assays were done initially at our research laboratory, and the assays at the research laboratory were cross‐validated with a national commercial laboratory (NMS). Validation parameters for assays at these laboratories were within the US Food and Drug Administration bioanalytical standards. These previously published findings confirmed the comparability of HCQ blood levels across laboratories included in this data set.^6^ HCQ blood level measurements are now commercially available through leading national laboratories (NMS, Exagen, LabCorp, ARUP, Mayo Clinic, etc)^25^, ^26^ and are covered by major payers under the Current Procedural Terminology code 80220 in the United States. Therefore, after the baseline visit, levels were measured through the clinical laboratories (NMS, the same laboratory that was used to validate our HCQ research assays initially) as part of routine care, and patients were asked to report any costs related with HCQ levels to the study team. All levels were measured in whole blood. Five visits reported HCQ serum levels that were converted to whole blood levels using the following formula: serum levels/0.53.^27^

### Data processing

We first calculated the weight‐based HCQ dose (mg/kg/day) for each patient at each visit using the following formula: HCQ dose at the visit/body weight at the visit. Weight‐based HCQ dose was used as a continuous variable in the analyses. Next, HCQ whole blood levels were used as continuous variables in the analyses. Given that some patients had HCQ levels <200 ng/mL, indicating severe nonadherence and likely missed doses ≥24 hours prior, we categorized HCQ blood levels into four groups for analysis: very low (<200 ng/mL; severe nonadherence), subtherapeutic (200 to <750 ng/mL; partial adherence), therapeutic (750 to 1,149 ng/mL), and supratherapeutic (≥1,150 ng/mL; potentially toxic).^6^, ^17^ Other variables including sex, race, insurance status were used as categorical variables. Medications were categorized as HCQ monotherapy (none) vs conventional disease‐modifying agents (cs‐DMARDs) vs biologics vs combination (biologic and cs‐DMARDs), prednisone >5 mg/d vs ≤5 mg/d, and CYP inhibitor use (yes or no). Variables such as age, eGFR, AST, and ALT were used as continuous variables. Additionally, CKD stages (CKD stage 1: ≥90, CKD stage 2: 89–60, CKD Stage 3 or above: <60) were determined using eGFR at the visit.^23^ Additionally, eGFR categories in 15‐unit increments (≥90, 89 to >75, 75 to >60, 60 to >45, 45 to >30, vs ≤30 mL/min/1.73 m^2^) were evaluated to better understand the eGFR threshold associated with higher HCQ blood levels. Chronic transaminitis was defined as present if a patient had two or more visits with abnormal AST and ALT (defined as >45 in our EHR) at least three to six months apart, which is a cut‐off commonly used by major laboratories as the upper normal limit. We intended to use advanced liver disease or cirrhosis to identify those with chronic liver disease, but the sample size was limited (n = 2 with cirrhosis).^28^ Gastric disease was defined as present if there was an active diagnosis on the problem list (peptic ulcer disease or gastritis), if the patient had an active prescription for gastric medicines for more than three months (eg, pantoprazole, famotidine), or if the patient had a gastric procedure documented in the EHR (eg, gastric sleeve, gastric bypass).

Finally, <5% of data were missing, and we imputed median values for laboratory or medication data and other variables from previous visits of the individual patient to handle missing data. When the outcome variable was missing, those patients were censored from the analysis (n = 1 patient with three visits).

### Analysis

Descriptive statistics were used to summarize baseline characteristics at index clinic visit as counts (percentages) for categorical variables and mean ± SD or median (range) for continuous variables. We also summarized visit‐level data.

Our conceptual model (Supplementary File 2) illustrates clinical factors, such as kidney clearance, gastric absorption, CYP inhibitors, patient‐reported adherence, and hepatic metabolism, which may influence variability in HCQ blood levels and that are not accounted for during routine HCQ dosing. We developed the generalized linear mixed models (GLMMs) to examine longitudinal associations between HCQ dose and HCQ blood levels in patients with impaired clearance (CKD), metabolism (chronic transaminitis, CYP inhibitors), or absorption (gastric diseases or medicines) adjusting for other covariables and adherence. The adjusted GLMMs were used to develop dose‐response relationship curves to compare the predicted HCQ blood levels with each 1 mg/kg/day increase in HCQ dose stratified by CKD, chronic transaminitis, and gastric disease categories (factors that could affect HCQ dose absorption and bioavailability in blood).^29^ GLMMs are unique because they incorporate both fixed and random effects, allowing the model to account for repeated measures at an individual patient level. Additionally, the GLMM models allow accounting for within‐subject variability in HCQ blood levels and other factors, such as HCQ dose, at each visit to inform predictions of an outcome over time. The flexibility of these models provides accurate and more robust estimates of longitudinal associations between two variables accounting for both within (intra‐) and between (inter‐) patient variations.^29^, ^30^

Next, we used GLMMs to examine longitudinal associations between active SLE, defined as SLEDAI‐2K ≥6,^20^ and HCQ whole blood level categories (very low, subtherapeutic, therapeutic, supratherapeutic) over time using a random slope and intercept model. In an additional longitudinal analysis, we used GLMMs to evaluate the odds of active SLE according to HCQ blood level maintenance patterns over follow‐up, with patients who remained persistently subtherapeutic (<750 ng/mL at all visits) serving as the reference group. These patients were compared with those who maintained therapeutic levels (≥750 ng/mL at all visits) or with those who dropped from therapeutic to subtherapeutic across visits. As a sensitivity analysis to the HCQ blood level category model, we applied a within‐between decomposition approach to partition time‐varying HCQ dose and blood levels into between‐patient means and within‐patient deviations to determine whether associations with active SLE were driven by stable inter‐individual differences or intra‐individual changes over time. We further applied this decomposition framework to examine associations between within‐ and between‐patient changes in continuous HCQ blood levels and both SLE disease activity and flares. GLMMs included random intercepts and random slopes for each participant, using an unstructured variance‐covariance matrix to account for the correlation between these random effects.^30^, ^31^

## RESULTS

### Patient characteristics

A total of 247 patients with SLE contributed 962 visits with paired random HCQ blood levels and clinical data. Average visits and follow‐up duration were four visits per patient and two years, respectively. At baseline, the mean ± SD patient age was 47 ±17 years, 91% were female, 66% were White, 14% were Black or African‐American, 9% were Asian, 9% were of Hispanic ethnicity, eGFR was 89 ± 27.1 mL/min/1.73 m^2^, 46% had HCQ blood levels <750 ng/mL (subtherapeutic), and 26% received an HCQ dose of >5 mg/kg/day (Table 1).

**Table 1:** Summary of patient and visit characteristics*

Characteristic	Value
Baseline clinic visit data (N = 247)	
Age at enrollment first visit in years	46.7 ± 16.7
Female	224 (91)
White	163 (66)
Black or African‐American	35 (14)
Asian	23 (9)
Hispanic or other ethnicity	23 (9)
Declined to report	3 (1)
Insurance type	
Private or commercial	172 (70)
Medicaid	39 (16)
Medicare	28 (11)
None	8 (3)
HCQ whole blood levels (categorical)	
<200 ng/mL	29 (12)
200 to <750 ng/mL	84 (34)
750–1,149 ng/mL	77 (32)
≥1,150 ng/mL	57 (23)
HCQ whole blood level in ng/mL (continuous)	837 ± 523
HCQ daily dose in mg/day	326 ± 90.5
Weight‐based HCQ dose in mg/kg/day	4.25 ± 1.19
Weight‐based HCQ dose >5 mg/kg/day	63 (26)
Liver function tests	
AST	23.6 ± 10.2
ALT	21.9 ± 13.9
eGFR (mL/min/1.73 m^2^)	89 ± 27.1
eGFR categories	
eGFR ≥90 mL/min/1.73 m^2^	139 (56)
eGFR 89 to >75 mL/min/1.73 m^2^	36 (15)
eGFR 75 to >60 mL/min/1.73 m^2^	44 (18)
eGFR 60 to >45 mL/min/1.73 m^2^	11 (4)
eGFR 45 to >30mL/min/1.73 m^2^	8 (3)
eGFR ≤30 mL/min/1.73 m^2^	9 (4)
Active proteinuria with low eGFR	10 (4)
Gastric disease	163 (66)
HCQ adherence (PDC)	90 ± 27%
SDI	0.98 ± 1.54
SLEDAI‐2K	3.25 ± 4.43
Other SLE medicines	
None	118 (48)
Disease‐modifying agents (cs‐DMARDs)	92 (38)
Biologics	17 (7)
Combination (cs‐DMARDs + biologics)	17 (7)
Prednisone dose, mg	7.39 ± 65.3
Visit‐level data (N = 962)	
Total number of visits	3.9 ± 2.18
Follow‐up length in days	714 ± 514
Median time between visits in days, median (IQR)	189 (104–407)
Insurance type	
Private or commercial	558 (58)
Medicaid	107 (11)
Medicare	190 (20)
None	107 (11)
HCQ adherence (PDC)	88.4 ± 23.38
HCQ whole blood levels (categorical)	
<200 ng/mL	97 (10)
200 to <750 ng/mL	360 (37)
750–1,149 ng/mL	301 (31)
≥1,150 ng/mL	204 (21)
HCQ whole blood level in ng/mL (continuous)	815 ± 482
Weight‐based HCQ dose (≤5 mg/kg/day), yes	761 (79)
Weight‐based HCQ dose, mg/kg/day	4.25 ± 1.07
Liver function tests	
AST	24.5 ± 12.5
ALT	22.4 ± 19.5
eGFR, mL/min/1.73 m^2^	88 ± 27.3
eGFR categories	
eGFR ≥90 mL/min/1.73 m^2^	525 (55)
eGFR 89 to >75 mL/min/1.73 m^2^	157 (16)
eGFR 75 to >60 mL/min/1.73 m^2^	143 (15)
eGFR 60 to >45 mL/min/1.73 m^2^	63 (7)
eGFR 45 to >30 mL/min/1.73 m^2^	39 (4)
eGFR ≤30 mL/min/1.73 m^2^	35 (4)
Gastric disease, yes	665 (70)
CYP inhibitors used, yes	606 (63)
Other SLE medicines	
None	390 (41)
Disease‐modifying agents (cs‐DMARDs)	387 (41)
Biologics	80 (8)
Combination (cs‐DMARDs + biologics)	96 (10)
Steroid dose >5 mg/day	94 (10)
Prednisone (mg/day)	6.78 ± 65.9
SLE disease outcomes	
SDI, mean ± SD	1.04 ± 1.41
SLE Disease Activity Index (SLEDAI‐2K)	2.76 ± 3.47
Active SLE (SLEDAI‐2K ≥6), yes	182 (19)
SLE flare, yes	99 (10)
HCQ related eye toxicity, yes	2 (1)^a^

* Data are presented as n (%) or mean ± SD unless otherwise specified. Baseline prednisone dose includes both maintenance oral therapy and acute pulse dosing where applicable. ALT, alanine aminotransferase; AST, aspartate aminotransferase; CKD‐EPI, Chronic Kidney Disease Epidemiology Collaboration; cs‐DMARDs, conventional synthetic disease‐modifying antirheumatic drugs; CYP, cytochrome P450; eGFR, estimated glomerular filtration rate; HCQ, hydroxychloroquine; IQR, interquartile range; PDC, proportion of days covered; SDI, Systemic Lupus International Collaborating Clinics Damage Index; SLE, systemic lupus erythematosus; SLEDAI‐2K, Systemic Lupus Erythematosus Disease Activity Index 2000.

^a^ 247 patients used as the denominator. eGFR was estimated per the 2021 CKD‐EPI equation.

Across visits (N = 962), the mean eGFR, HCQ dose, and HCQ blood levels were 88.2 ± 27.3 mL/min/1.73 m^2^, 4.25 ± 1.07 mg/kg/day, and 815 ± 482 ng/mL, respectively. Patients during a total of 761 visits (79.1%) received a weight‐based HCQ dose of ≤5 mg/kg/day; 10%, 37%, 31%, and 21% visits had very low (<200 ng/mL), subtherapeutic or intra‐therapeutic (<750 ng/mL), therapeutic (750–1,150 ng/mL), and supratherapeutic (≥1,150 ng/mL) levels, respectively. An equal distribution of repeated HCQ blood level measurements was seen in the insurance category, with 58% visits in private or commercial insurance and 42% with public (Medicaid or Medicare) or no insurance (Table 1). Only two patients with commercial insurance reported a co‐pay of $50 to $100 for HCQ blood level testing. Mean adherence and mean change in HCQ blood levels between visits by HCQ blood level categories are shown in Supplementary File 3.

### Relationship between HCQ dose and HCQ levels stratified by CKD stage categories, chronic transaminitis, and gastric disease

Given that we collected HCQ blood levels at least six hours from the last HCQ dose, thus avoiding peak HCQ blood levels, we used the mid‐point of the 8% to 27% within‐day variability range (15%) to identify factors associated with clinically meaningful changes in HCQ blood levels (which will be 122 ± 65 ng/mL based on our mean levels of 815 ± 482 ng/mL)^32^, ^33^, ^34^ using adjusted GLMM models with random intercept and slope. We noted a linear association between HCQ dose and HCQ blood levels. In particular, after stratifying by CKD stage categories, we noted that patients with CKD stage ≥3 had significantly higher HCQ blood levels by 213.2 ± 63.7 ng/mL (*P* = 0.001) (Supplementary Files 4 and 5) despite receiving a weight‐based HCQ dose. However, patients with CKD stage 2 had higher predicted change in HCQ blood levels by 92.5 ± 39.2 ng/mL (*P* value = 0.02) (Supplementary Files 4 and 5). Because of significant differences in HCQ levels between CKD stage 2 and CKD stage ≥3, we used the eGFR categories in 15‐unit increments: ≥90, 89 to >75, 75 to >60, 60 to >45, 45 to >30, and ≤30 mL/min/1.73 m^2^. Patients with eGFR categories 75 to >60, 60 to >45, 45 to >30, and ≤30 mL/min/1.73 m^2^ had mean HCQ levels 200.1 ± 47.6 ng/mL (*P* value <0.0001), 179.6 ± 69.7 ng/mL (*P* = 0.01), 275.7 ± 89.0 ng/mL (*P* = 0.002), and 479.0 ± 92.2 ng/mL (*P* value <0.0001) higher, respectively, compared with eGFR ≥90 mL/min/1.73 m^2^ (Table 2). More importantly, as shown in Figure 1, even in patients with eGFR ≤75, a weight‐based HCQ dose of 5 mg/kg/day predicted supratherapeutic HCQ blood levels (≥1,150 ng/mL), increasing the risks of toxicity over time.

**Table 2:** Multivariable generalized linear mixed‐effects model showing the predicted change in HCQ blood levels using eGFR categories in 15‐unit increments in patients with SLE (n = 962 visits)*

Variables	Estimated change in HCQ blood levels, beta coefficient ± SE, ng/mL	*P* value
Age, per 1‐year increase	0.13 ± 1.71	0.94
Male	Ref	−
**Female**	**−182.8 ± 76.7**	**0.02**
White	Ref	−
Black or African‐American	24.1 ± 69.5	0.73
**Asian**	**−168.2 ± 79.2**	**0.03**
Hispanic or other ethnicity^a^	−64.8 ± 71.4	0.36
Insurance type or status		
Private or commercial, yes	Ref	−
Medicare, yes	−45.4 ± 63.8	0.48
Medicaid, yes	−9.8 ± 43.2	0.82
No insurance	43.5 ± 42.1	0.30
eGFR categories		
eGFR ≥90 mL/min/1.73 m^2^	Ref	−
eGFR 89 to >75 mL/min/1.73 m^2^	33.5 ± 39.0	0.39
**eGFR 75 to >60 mL/min/1.73 m** ^**2**^	**200.1 ± 47.6**	**<0.0001**
**eGFR 60 to >45 mL/min/1.73 m** ^**2**^	**179.6 ± 69.7**	**0.01**
**eGFR 45 to >30mL/min/1.73 m** ^**2**^	**275.7 ± 89.0**	**0.002**
**eGFR ≤30 mL/min/1.73 m** ^**2**^	**479.0 ± 92.2**	**<0.0001**
Persistent liver enzyme elevation (chronic transaminitis) status		
Chronic transaminitis, no	Ref	−
Chronic transaminitis, yes	66.4 ± 74.1	0.37
Gastric disease status		
Gastric disease, no	Ref	−
Gastric disease, yes	−21.2 ± 49.2	0.67
**CYP inhibitors used, yes**	**82.1 ± 38.7**	**0.04**
PDC per 1% increase	−0.7 ± 0.6	0.21
Other SLE medicines		
None	Ref	−
Disease‐modifying agents (cs‐DMARDs), yes	7.5 ± 36.5	0.84
Biologics, yes	3.4 ± 56.4	0.95
Combination, yes	−17.3 ± 54.9	0.75
**Weight‐based HCQ dose per 1 mg/kg/day increments**	**96.7 ± 15.4**	**<0.0001**

* One patient with no outcome variable data with three visits was excluded. Values represent the estimated change in hydroxychloroquine (HCQ) blood levels (ng/mL) (beta coefficient ± SE), adjusted for all variables in the model. *P* value < 0.05 indicate statistical significance shown in bold font. A positive value indicates higher HCQ levels compared with the reference group. cs‐DMARDs, conventional synthetic disease‐modifying antirheumatic drugs; CYP, cytochrome P450; eGFR, estimated glomerular filtration rate; HCQ, hydroxychloroquine; PDC, proportion of days covered; SLE, systemic lupus erythematosus.

^a^ Includes visits that did not report race or ethnicity.

**Figure 1:**
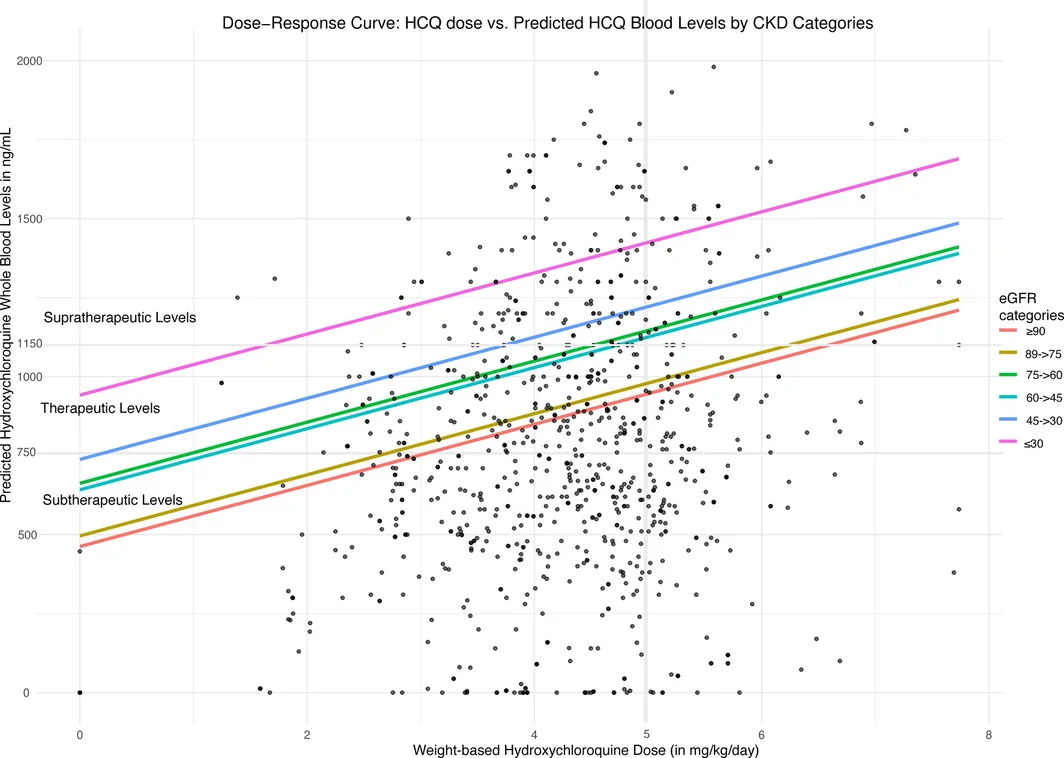
Dose‐response curves between HCQ weight‐based dose (mg/kg/day) and HCQ blood levels (ng/mL) over time in patients using eGFR categories in 15 units increments in patients with SLE. CKD, chronic kidney disease; eGFR, estimated glomerular filtration rate; HCQ, hydroxychloroquine; SLE, systemic lupus erythematosus.

Next, no significant changes in HCQ blood levels were predicted in those with chronic transaminitis compared with those without chronic transaminitis with same weight‐based HCQ dose (Supplementary File 5); the predicted HCQ blood level variations by chronic transaminitis were 66.4 ± 74.1 ng/mL (*P* value = 0.37) (Table 2). However, the presence of gastric disease did not predict significant variations in HCQ whole blood levels with weight‐based HCQ dosing (−21.2 ± 49.2 ng/mL) (Table 2, Supplementary File 6). HCQ blood levels were lower by 182 ng/mL in women and by 168 ng/mL in Asian patients (Table 2). Finally, CYP inhibitor use was linked with slightly higher HCQ blood levels by 82.1 ng/mL, indicating a mild‐moderate effect on HCQ metabolism and levels (Table 2).

### Therapeutic HCQ whole blood levels predict lower odds of active SLE (SLEDAI‐2K ≥ 6) over time

Using the GLMMs with random intercepts and random slopes, we examined how maintaining HCQ blood levels in the therapeutic range (750–1,149 ng/mL) predicted active SLE (SLEDAI‐2K ≥6) over longitudinal follow‐up. First, continuous HCQ blood levels were associated with active SLE; for every 100 ng/mL increase in HCQ blood levels over time, the odds of active SLE (SLEDAI‐2K ≥6) decreased by 12% (odds ratio [OR] 0.88, 95% confidence interval [CI] 0.81–0.95, *P* value = 0.002) (Supplementary Files 7 and 8). Next, using HCQ blood level categories (therapeutic, subtherapeutic, and supratherapeutic levels with the therapeutic levels as the reference), subtherapeutic HCQ levels (200 to <750 ng/mL) predicted a 2.6‐fold higher odds of active SLE (OR 2.57, 95% CI 1.17–5.70, *P* value = 0.02) (Table 3). Very low HCQ levels (<200 ng/mL) predicted 6.7‐fold higher odds of active SLE (OR 6.65, 95% CI 2.07–21.36, *P* value = 0.001) (Table 3). Supratherapeutic levels (≥1,150 ng/mL) did not significantly predict lower SLE disease activity compared with therapeutic levels (OR 0.63, 95% CI 0.25–1.64, *P* value = 0.35). A higher weight‐based HCQ dose per 1 mg/kg/day increment was associated with increased odds of active SLE (OR 1.54, 95% CI 1.05–2.25, *P* = 0.02) (Table 3). Similarly, cs‐DMARD therapy (OR 4.83, 95% CI 2.1–11.3, *P* < 0.001) (Table 3) and prednisone use >5 mg/day (OR 11.64, 95% CI 4.1–33.3, *P* < 0.001) (Table 3) were associated with greater odds of active SLE, which likely reflects confounding by indication because patients with more active disease may receive treatment intensification.

**Table 3:** Generalized linear mixed‐effects model using random slopes and intercepts showing predictors of active SLE (SLEDAI‐2K ≥6) over time in SLE using HCQ whole blood levels categories (N = 962 visits)*

Variables at visit	Adjusted OR (95% CI)	*P* value
Age, per 1‐year increase	0.97 (0.93–1.00)	0.09
Male	Ref	−
Female	0.37 (0.08–1.85)	0.23
White	Ref	−
Black or African‐American	0.52 (0.12–2.27)	0.38
Asian	1.81 (0.39–8.50)	0.45
Hispanic or other ethnicity^a^	2.43 (0.63–9.36)	0.20
Insurance type or status		
Private or commercial, yes	Ref	−
Medicaid, yes	0.18 (0.03–1.18)	0.07
Medicare, yes	0.59 (0.22–1.61)	0.30
No insurance	1.06 (0.41–2.79)	0.90
PDC per 1% increase	1.00 (0.99–1.01)	0.74
HCQ whole blood levels (categorical) −		
**HCQ levels <200 (very low)**	**6.65 (2.07–21.36)**	**0.001**
**HCQ levels 200–<750 (subtherapeutic levels)**	**2.57 (1.17–5.70)**	**0.02**
HCQ levels 750–1,149 (therapeutic levels)	Ref	−
HCQ levels ≥1,150 (supratherapeutic levels)	0.63 (0.25–1.64)	0.35
**Weight‐based HCQ dose per 1 mg/kg/day increments**	**1.54 (1.05–2.25)**	**0.02**
Other SLE medicines		
None	Ref	−
**Disease‐modifying agents (cs‐DMARDs), yes**	**4.83 (2.06–11.34)**	**<0.001**
Biologicals, yes	0.98 (0.29–3.33)	0.98
Combination, yes	1.65 (0.53–5.15)	0.39
**Prednisone dose >5 mg, yes**	**11.64 (4.06–33.33)**	**<0.001**
**eGFR per 15 mL/min/1.73 m** ^**2**^ **increase**	**0.73 (0.56–0.97)**	**0.03**
SDI per 1‐unit increase	1.13 (0.85–1.51)	0.37

* One patient with no outcome variable data with three visits was excluded. Values represent adjusted OR with 95% CIs, derived from a GLMM with random slopes and intercepts, adjusted for all variables in the model. *P* values < 0.05 indicate statistical significance shown in bold font. A value < 1.0 indicates lower odds of active SLE compared with the reference group, while a value > 1.0 indicates higher odds. + indicates trend toward significance. CI, confidence interval; cs‐DMARDs, conventional synthetic disease‐modifying antirheumatic drugs; eGFR, estimated glomerular filtration rate; GLMM, generalized linear mixed model; HCQ, hydroxychloroquine; OR, odds ratio; PDC, proportion of days covered; SDI, Systemic Lupus International Collaborating Clinics Damage Index; SLE, systemic lupus erythematosus; SLEDAI‐2K, Systemic Lupus Erythematosus Disease Activity Index 2000.

^a^ Includes visits that did not report race or ethnicity.

Patients who maintained HCQ levels ≥750 ng/mL throughout all visits had 83% lower odds of active SLE (OR 0.17, 95% CI 0.04–0.75, *P* value = 0.02) (Table 4) compared with patients who maintained subtherapeutic levels (<750 ng/mL). In contrast, patients who transitioned from therapeutic to subtherapeutic levels did not demonstrate significantly different odds of active SLE compared with the persistently subtherapeutic group (OR 0.50, 95% CI 0.14–1.75, *P* = 0.28) (Table 4).

**Table 4:** Generalized linear mixed‐effects model showing adjusted odds of active SLE (SLEDAI‐2K ≥6) according to longitudinal HCQ blood level maintenance patterns over time in SLE (n = 962 visits)*

Variables at visit	Adjusted OR (95% CI)	*P* value
**Age, per 1‐year increase**	**0.94 (0.90–0.98)**	**0.007**
Male	Ref	−
Female	0.27 (0.04–1.67)	0.16
White	Ref	−
Black or African‐American	0.31 (0.06–1.61)	0.16
Asian	1.10 (0.20–5.89)	0.91
Hispanic or other ethnicity^a^	1.90 (0.43–8.31)	0.40
Insurance type or status		
Private or commercial	Ref	−
Medicaid	0.77 (0.12–4.92)	0.78
Medicare	0.87 (0.31–2.47)	0.80
No insurance	1.57 (0.59–4.19)	0.37
Patient‐reported adherence per 1% increase	1 (0.99–1.02)	0.70
eGFR per 15 mL/min/1.73 m^2^ increase	0.87 (0.66–1.14)	0.32
SDI per 1‐unit increase	1.29 (0.94–1.76)	0.11
HCQ levels over time		
HCQ levels remains <750 ng/mL	Ref	−
**HCQ levels ≥750 ng/mL all visits**	**0.17 (0.04–0.75)**	**0.02**
HCQ levels drop <750 ng/mL	0.50 (0.14–1.75)	0.28
Weight‐based HCQ dose per 1 mg/kg/day increments	1.41 (0.96–2.09)	0.08
Other SLE medicines		
None	Ref	−
**Disease‐modifying agents (cs‐DMARDs), yes**	**3.41 (1.41–8.22)**	**0.006**
Biologics, yes	1.14 (0.33–3.91)	0.84
Combination, yes	1.20 (0.36–4.05)	0.76
**Prednisone dose >5 mg, yes**	**14.40 (4.57–45.60)**	**<0.001**

* One patient with no outcome variable data with three visits was excluded. Values represent adjusted OR with 95% CIs, derived from a GLMM with random intercepts and slopes, adjusted for all covariates shown. HCQ blood level categories were defined longitudinally as maintained therapeutic (≥750 ng/mL at all visits), switched from therapeutic to subtherapeutic (<750 ng/mL at any visit), or persistently subtherapeutic (<750 ng/mL at all visits). A value <1.0 indicates lower odds of active SLE compared with the Reference group, while a value >1.0 indicates higher odds. *P* values <0.05 indicate statistical significance shown in bold font. CI, confidence interval; cs‐DMARDs, conventional synthetic disease‐modifying antirheumatic drugs; eGFR, estimated glomerular filtration rate; GLMM, generalized linear mixed model; HCQ, hydroxychloroquine; OR, odds ratio; SDI, Systemic Lupus International Collaborating Clinics Damage Index; SLE, systemic lupus erythematosus; SLEDAI‐2K, Systemic Lupus Erythematosus Disease Activity Index 2000.

^a^ Includes visits that did not report race or ethnicity.

### Sensitivity analysis: within‐ and between‐subject effects of HCQ dose and HCQ blood levels on active SLE (SLEDAI‐2K ≥ 6) over time

To specifically examine the within‐patient relationships and how changes in HCQ dose and HCQ blood levels within the same patient over time affect the outcome (active SLE), we used the within‐between subjects decomposition approach. Through this approach, we included changes in HCQ dose and blood levels over time at an individual patient level (within subjects) and mean differences among patients’ doses and blood levels (between subjects). This sensitivity analysis showed that every 100 ng/mL increase in HCQ blood levels at an individual patient level during follow‐up predicted 33% lower odds of active SLE (95% CI 0.52–0.86, *P* value = 0.002) (Supplementary File 9). Next, using HCQ blood levels categories and within and between subjects changes in weight‐based HCQ dose over time, supratherapeutic levels (≥1,150 ng/mL) did not significantly predict lower SLE disease activity (OR 0.68, 95% CI 0.28–1.68, *P* value = 0.41) (Supplementary File 10), indicating a therapeutic saturation effect. However, having subtherapeutic HCQ levels (200 to <750 ng/mL) predicted two‐fold higher odds of active SLE compared with having therapeutic levels (750 to 1,149 ng/mL) (OR 2.04, 95% CI 0.95–4.38, *P* = 0.07) (Supplementary File 10). Very low HCQ levels (<200 ng/mL) were associated with 4.8‐fold higher odds of active SLE (OR 4.76, 95% CI 1.56–14.51, *P* value = 0.006) (Supplementary File 10).

### Other outcomes: SLE flares and HCQ toxicity over time after baseline visit

A total of 99 visit‐level flares (10%) were noted with 30% visit‐level flares in patients with very low and subtherapeutic levels and 19% flares in those with therapeutic and supratherapeutic levels. Using the within‐between decomposition approach, a similar association between within subject HCQ blood levels increase and likelihood of SLE flare was noted where every 100 ng/mL increase in HCQ blood levels at each visit predicted 22% lower odds of SLE flare (OR 0.78, 95% CI 0.62–0.98, *P* value 0.03) (Table 5). Two patients who had levels >1,150 ng/mL at two visits despite weight‐based dosing were found to have preclinical eye toxicity after the second visit, resulting in discontinuation of HCQ.

**Table 5:** Generalized linear mixed‐effects model examining the between‐patient and within‐patient effects of HCQ dose and HCQ blood level on SLE flare over time after baseline visit (n = 962)*

Variables at visit	Adjusted OR (95% CI)	*P* value
Age, per 1‐year increase	0.99 (0.98–1.02)	0.73
Male	Ref	−
Female	0.86 (0.39,1.89)	0.70
White	Ref	−
Black or African‐American	0.84 (0.37–1.94)	0.69
Asian	1.91 (0.88–4.16)	0.10
Hispanic or other ethnicity^a^	2.1 (1.02, 4.4)	0.045
Insurance type or status		
**Private or commercial**	**2.11 (1.02–4.39)**	**0.044**
Medicaid	0.48 (0.17–1.42)	0.19
Medicare	0.70 (0.35–1.39)	0.31
No insurance	0.65 (0.28–1.48)	0.30
PDC per 1% increase	1.00 (0.99–1.01)	0.97
eGFR per 15 mL/min/1.73 m^2^ increase	0.90 (0.77–1.05)	0.17
SDI per 1‐unit increase	1.10 (0.91–1.31)	0.33
HCQ dose		
HCQ dose change per 1‐unit change (within‐patient effect)	1.17 (0.89–1.52)	0.26
Mean HCQ dose in mg/kg/day (between‐patient effect)	1.05 (0.82–1.35)	0.70
HCQ levels		
**Within patients HCQ blood levels increase per 100 ng/mL**	**0.78 (0.62–0.98)**	**0.03**
Between patients mean difference in HCQ levels in 100 ng/mL increments	0.95 (0.73–1.25)	0.72
Other SLE medicines		
None	Ref	−
Disease‐modifying agents (cs‐DMARDs), yes	1.49 (0.86–2.58)	0.16
Biologics, yes	1.19 (0.47–2.99)	0.71
Combination, yes	1.49 (0.65–3.41)	0.35
**Prednisone dose >5 mg, yes**	**2.88 (1.51–5.50)**	**0.0014**

* One patient with no outcome variable data with three visits was excluded. Values represent adjusted OR with 95% CI, derived from a GLMM with random slopes and intercepts, adjusted for all variables in the model. *P* values < 0.05 indicate statistical significance shown in bold font. A value < 1.0 indicates lower odds of active SLE compared with the reference group, whereas a value > 1.0 indicates higher odds. + indicates trend toward significance. CI, confidence interval; cs‐DMARDs, conventional synthetic disease‐modifying antirheumatic drugs; eGFR, estimated glomerular filtration rate; GLMM, generalized linear mixed model; HCQ, hydroxychloroquine; OR, odds ratio; PDC, proportion of days covered; SDI, Systemic Lupus International Collaborating Clinics Damage Index; SLE, systemic lupus erythematosus.

^a^ Includes visits that did not report race or ethnicity.

## DISCUSSION

Building on existing literature, this longitudinal study is the first to demonstrate that besides CKD stage ≥3, even in patients with eGFR 75 to >60 mL/min/1.73 m^2^, weight‐based HCQ dosing resulted in 200 ng/mL higher HCQ whole blood levels and predicted supratherapeutic levels ≥1,150 ng/mL over time, which could potentially accelerate toxicity risk. These findings underscore a need for close monitoring with HCQ blood levels in all patients with eGFR ≤75 mL/min/1.73 m^2^ to inform optimal HCQ dosing balancing safety vs efficacy in this population. Importantly, this study provides preliminary data reassuring that HCQ dose could be continued without adjustments in chronic transaminitis, and gastric disease through external validation is needed. Finally, this longitudinal study spanning over at least two years of follow‐up highlighted that subtherapeutic (200 to <750 ng/mL) and very low HCQ blood levels (<200 ng/mL) raised the odds of active SLE by 2.6‐fold and 6.7‐fold, whereas maintaining levels within the therapeutic range of 750 to <1,150 ng/mL over time predicted 83% lower odds of active SLE. These findings establish the potential clinical significance of routine HCQ blood level monitoring to guide optimal use of HCQ based on individual patient risk factors. Finally, these findings lay a roadmap for a future clinical trial comparing the safety and effectiveness of HCQ dosing informed by HCQ blood level monitoring to standard flat weight‐based dosing, particularly in high‐risk groups such as those with eGFR ≤75 mL/min/1.73 m^2^.

Previous studies were predominantly cross‐sectional and showed that impaired kidney function is associated with higher HCQ concentrations. One study reported median levels of 1,338 ng/mL in CKD stage ≥3 patients compared with 817 ng/mL without CKD,^35^ whereas another noted 2× higher HCQ blood levels in patients with vs without eGFR <60 (2,404 vs 1,046 ng/mL).^36^ Our study extends the previous cross‐sectional work by quantifying the change in HCQ blood levels by continuing the same dose in patients with normal and reduced kidney function (eGFR) accounting for repeated measurements and intra‐individual dose changes over time. Our study validates previous findings that despite weight‐based dosing, patients with CKD Stage 3 and above can have supratherapeutic HCQ blood levels. More importantly, our study highlights for the first time that even patients with some kidney function impairment (eGFR 75 to >60 mL/min/1.73 m^2^) face a significant increase in HCQ blood levels by 200 ng/mL over time beyond CKD stage ≥3 (eGFR ≤60 mL/min/1.73 m^2^). These findings show that continuing a weight‐based dose of 5 mg/kg/day without any adjustments for kidney function in patients with eGFR of ≤75 mL/min/1.73 m^2^ could be associated with supratherapeutic HCQ blood levels over time, increasing toxicity risk without statistically significant clinical benefit. A large cohort study highlighted that patients with CKD stage ≥3 face two‐fold higher HCQ retinopathy risk,^37^ and another study showed that arbitrary HCQ dose reductions^33^ in patients with CKD could raise the risk of active SLE or flares, supporting that our findings will have clear implications for clinical practice. This study provides critically needed data on how close monitoring via HCQ blood level monitoring could inform precise and more balanced HCQ dose in patients with impaired kidney function. Additionally, this study highlights that potentially even patients with eGFR 75 to 60 mL/min/1.73 m^2^ could benefit from close HCQ blood level monitoring to guide optimal HCQ use beyond CKD stage ≥3 (eGFR ≤60 mL/min/1.73 m^2^).

This longitudinal study shows that chronic transaminitis, gastric disease, or acid reducing medicine do not affect HCQ blood levels, although validation in other cohorts and in those clinical diseases (eg, cirrhosis) is needed. Interestingly, lower HCQ blood level concentrations were seen in women and Asian patients despite adjusting for weight‐based HCQ dose over time. Data on sex or ethnicity and HCQ blood levels have been conflicted. Although some studies report no associations, others note lower levels in women, and a few report higher levels and higher chances of toxicity due to differences in drug metabolism, higher doses, and estrogen's effect on CYP, which is a key HCQ metabolizer.^33^, ^36^, ^37^, ^38^ Pharmacogenetic variability may help explain our findings in Asian patients. For example, studies report that the CYP2D6*10 allele, which reduces CYP2D6 activity and alters HCQ metabolism, is more common in East Asians (~50%) than in Europeans (~1–4%).^39^, ^40^ Therefore, understanding associations between CYP metabolism and HCQ blood levels will be pivotal to inform optimal HCQ dosing in this population. Nevertheless, our findings underscore a potential role of HCQ blood level monitoring in bypassing clinical variables affecting HCQ absorption and metabolism and providing a true snapshot of actual HCQ exposure to inform optimal HCQ use at an individual patient level.

Finally, this study establishes the longitudinal impact of maintaining levels in the therapeutic range on SLE outcomes. Low HCQ levels are consistently linked to worse SLE outcomes, but previous studies have typically assessed levels at a single time point, used median or mean values across visits, or treated each measurement as an independent observation (Supplementary File 11).^14^, ^41^, ^42^, ^43^, ^44^, ^45^, ^46^, ^47^ Although these approaches have advanced understanding of the relationship between HCQ blood levels and SLE outcomes, additional insights on how HCQ blood level monitoring relates to SLE disease control at an individual patient level over time are needed. This study directly addresses these gaps because it leverages GLMM models that predict the risk of active SLE (SLEDAI‐2K ≥6) and flares over time, accounting for the repeated HCQ blood level measurements, HCQ dose, and other values. Using the within‐between subjects decomposition approach, we found that an increase in an individual patient's HCQ blood levels by 100 ng/mL at each follow‐up reduced the risk of active SLE by 33% and lowered SLE flare risk by 22%. Our longitudinal analysis showed that subtherapeutic (200 to <750 ng/mL) and very low levels (<200 ng/mL) predicted a 2.6‐fold and 6.7‐fold higher odds of active SLE, respectively. Having supratherapeutic levels (≥1,150 ng/mL) did not lower the odds of active SLE but accelerated toxicity despite weight‐based dosing. A key finding of this work included data showing that patients maintaining levels within the therapeutic range (750 to <1,150 ng/mL) had 83% lower odds of active SLE compared with patients who had subtherapeutic levels (<750 ng/mL) across visits. Collectively, our findings, along with previous cross‐sectional data, support the clinical relevance of HCQ blood level monitoring and highlight the need for prospective interventional studies to determine whether routine level‐guided dose adjustments could ensure better SLE outcomes and minimize toxicity.^8^, ^12^, ^13^, ^14^, ^15^, ^18^, ^27^, ^33^, ^35^, ^43^, ^48^, ^49^, ^50^, ^51^ Our study also provides real‐world evidence that HCQ blood level monitoring was covered by most private and public insurers in the United States, given that only two patients had a co‐pay of $50 to $100 for HCQ blood levels, addressing the prevailing concerns around cost and coverage.

This study has several strengths, including longitudinal data collection spanning over three years and at least four visits with HCQ blood level measurements per patient. However, we do acknowledge limitations. Although prospective and longitudinal in design, it was conducted in a single academic center, which may limit the generalizability of our findings to all SLE populations. Second, our study could not compare fluctuations in HCQ blood levels in patients with acute kidney injury (AKI) vs CKD. Although our cohort included 10 patients with active proteinuria and low eGFR, their eGFR values remained stable throughout follow‐up, suggesting chronic kidney impairment rather than AKI. Third, our models adjusted for CYP inhibitors but not for CYP metabolism. Given pharmacogenetic variability in Asian populations, future analysis should adjust for CYP metabolism to quantify differences in blood concentrations of HCQ to inform optimal HCQ use in such groups. Fourth, we categorized immunosuppressants as DMARDs, biologics, or combination to examine time‐varying effects of change in immunosuppressants status. However, this categorization could have obscured clinical effects resulting from switching between medicines within the same immunosuppressants category (eg, methotrexate to mycophenolate) or steroid taper regimens in‐between visits. Fifth, qualitative assessments of patient and clinician experiences with HCQ level testing are underway to guide open communication regarding HCQ blood level monitoring, and a cost‐benefit analysis accounting for patient‐reported adherence is required.^52^ Moreover, we observed high mean PDC even among patients with very low HCQ levels of <200 ng/mL, raising the possibility that pharmacy refill‐based adherence measures and HCQ blood levels may capture different aspects of medication use, reflecting longer‐term refill patterns versus more recent drug exposure.^13^ Finally, it is possible that enrollment in a longitudinal observational cohort improved behavior and thereby increased adherence, similar to the findings of the Plaquenil Lupus Systemic study.^14^ A mixed methods implementation study that systematically tests the safety and efficacy of integrating HCQ blood level monitoring to inform optimal HCQ dosing and improve adherence trends over time in clinical care compared with the old “one‐size‐fits‐all” weight‐dosing is the next logical step to provide precise and personalized care at the individual patient level in SLE.

In summary, the impact of this study is four‐fold. First, it provides longitudinal evidence that having subtherapeutic (200 to <750 ng/mL) and very low levels (<200 ng/mL) can predict 2.6‐fold and 6.7‐fold higher odds of active SLE, that increasing HCQ levels by 100 ng/mL at each visit could lower odds of active SLE by 33% and risk of flares by 22%, and maintaining levels in therapeutic range can lower odds of active SLE by 83%. Second, this study highlights that HCQ blood level monitoring has the potential to be a practical tool that could optimize HCQ dosing at the individual patient level, bypassing variables that affect absorption and clearance of HCQ. Third, this study provides longitudinal evidence on groups, such as eGFR (≤75 mL/min/1.73m^2^), which are at higher risk of supratherapeutic and potentially toxic levels despite weight‐based dosing, thereby warranting close monitoring via HCQ blood level measurements to guide optimal HCQ use. Finally, this study provides real‐world US data on the feasibility of using HCQ blood levels in regard to insurance coverage and costs as part of routine SLE care in the United States.

## AUTHOR CONTRIBUTIONS

All authors contributed to at least one of the following manuscript preparation roles: conceptualization AND/OR methodology, software, investigation, formal analysis, data curation, visualization, and validation AND drafting or reviewing/editing the final draft. As corresponding author, Dr Garg confirms that all authors have provided the final approval of the version to be published and takes responsibility for the affirmations regarding article submission (eg, not under consideration by another journal), the integrity of the data presented, and the statements regarding compliance with institutional review board/Declaration of Helsinki requirements.

## Supporting information


**Disclosure Form**:

**Data S1.** Supporting Information.
