# Supplementary material for: Real‐World Longitudinal Data on the Impact of Hydroxychloroquine Blood Level Monitoring on Lupus Outcomes: Results of a Prospective Longitudinal Cohort Study
Source: Arthritis Care Res (Hoboken). 2026 May 3;78(11):1618–30. doi: 10.1002/acr.80059 (PMC13463390; doi:10.1002/acr.80059)
Supplement: Supplementary file 2 [file ACR-78-1618-s002.docx]

**Supplementary File 1. Common CYP Inhibitors of Hydroxychloroquine Metabolism**

**CYP2D6 inhibitors**

**Strong inhibitors:** Fluoxetine, bupropion, paroxetine

**Weak inhibitors:** amiodarone, celecoxib, cimetidine, sertraline, labetalol, escitalopram

**CYP2C8 inhibitors**

Montelukast, gemfibrozil, clopidogrel, salmeterol, felodipine
**CYP3A4 inhibitors**

Ketoconazole, clarithromycin, diltiazem, lopinavir (and other HIV protease inhibitors), tamoxifen, verapamil

**Supplementary File 2. A Conceptual Model of Clinical Factors that Could Potentially Influence Hydroxychloroquine Levels**

| **Supplementary File 3. Mean Change in HCQ Blood Levels Between Visits and Mean PDC/Adherence by Hydroxychloroquine Blood Level Categories** | | |
| --- | --- | --- |
| **HCQ Blood Level Category (ng/mL)** | **Mean Change in HCQ Level Between Visits (ng/mL), Mean ± SD** | **Mean PDC/Adherence (%), Mean ± SD** |
| <200 | -149 ± 73 | 83.6 ± 29.1 |
| 200-<750 | -0.5 ± 51 | 89.7 ± 20.9 |
| 750 - 1149 | 35 ± 60 | 88.6 ± 23.7 |
| ≥ 1150 | 110 ± 75 | 88.3 ± 23.8 |

**Supplementary File 4: Multivariable Generalized Linear Mixed-Effects Model Showing the Predicted Change in HCQ Blood Levels with Different Variables Over Time in Patients with SLE (n=962 visits*)**

| **Variables** | **Change in HCQ Level ng/ml**  **(Mean ± SD)** | **p-value** |
| --- | --- | --- |
| Age, per 1-Year Increase | 0.05±1.89 | 0.98 |
| Sex |  |  |
| Male | ref |  |
| **Female** | **-199.0 ± 80.0** | **0.01** |
| Race and Ethnicity |  |  |
| White | ref |  |
| Black or African American | 21.9 ± 74.4 | 0.77 |
| **Asian** | **-207.7 ± 89.1** | **0.02** |
| Hispanic or Other Ethnicity** | -38.9 ± 79.9 | 0.63 |
| Insurance Type or Status |  |  |
| Private or Commercial, yes | ref |  |
| Medicare, yes | -77.8 ± 68.9 | 0.26 |
| Medicaid, yes | 15.5 ± 45.8 | 0.73 |
| No Insurance | 73.0 ± 46.3 | 0.12 |
| **Chronic Kidney Disease (CKD) Stage** |  |  |
| CKD Stage 1 (eGFR ≥90), yes | ref |  |
| **CKD Stage 2 (eGFR 89-60), yes** | **92.5 ± 39.2** | **0.02** |
| **CKD Stage ≥3 (eGFR <60), yes** | **213.2 ± 63.7** | **0.001** |
| Persistent Liver Enzyme Elevation (PLEE) Status |  |  |
| PLEE, no | ref |  |
| PLEE, yes | 82.9 ± 77.8 | 0.29 |
| Gastric Disease Status |  |  |
| Gastric Disease, no | ref |  |
| Gastric Disease, yes | -66.7 ± 53.7 | 0.22 |
| **CYP Inhibitors Used, yes** | **106.2 ± 41.8** | **0.01** |
| Patient-Reported Adherence per 10% Increase | -9.3 ± 5.9 | 0.12 |
| Other SLE Medicines |  |  |
| None | ref |  |
| Disease Modifying Agents (cs-DMARDs), yes | -6.0 ± 40.4 | 0.88 |
| Biologics, yes | -3.75 ± 60.4 | 0.95 |
| Combination, yes | -90.1 ± 59.3 | 0.13 |
| **Weight-based HCQ Dose per 1 mg/kg/day Increments** | **89.5 ± 16.7** | **<0.0001** |

Footnotes: Values represent the mean change in hydroxychloroquine (HCQ) blood level (ng/mL) ± standard deviation, adjusted for all variables in the model. P values < 0.05 indicate statistical significance. A positive value indicates higher HCQ levels compared with the reference group. Abbreviations: CKD = chronic kidney disease; PLEE = persistent liver enzyme elevation; cs-DMARDs = conventional synthetic disease-modifying antirheumatic drugs; CYP = Cytochrome P450. *1 patient with no outcome variable data with 3 visits was excluded. **Includes visits that did not report race or ethnicity.

**Supplementary File 5. Dose-Response Curves Between HCQ Weight-Based Dose (mg/kg/d) and HCQ Blood Levels (ng/mL) Over Time in Patients with Chronic Kidney Disease Stage 1, 2, ≥3 (Panel A), and Chronic transaminitis (Panel B).**

**Supplementary File 6. Dose-Response Curves Between HCQ Weight-Based Dose (mg/kg/d) and HCQ Blood Levels (ng/mL) Over Time in Patients With Gastric Disease or Gastric Surgery or on Chronic Acid-Reducing Medicines**

**Supplementary File 7:** Generalized Linear Mixed Effects Model Using Random Slopes and Intercepts Showing Predictors of Active SLE (SLEDAI-2K ≥6) Over Time in SLE (n=962*)

| **Variables at Visit** | **Adjusted Odds Ratio (95% CI)** | **p-value** |
| --- | --- | --- |
| **Age, per 10-Year Increase** | **0.95 (0.91, 0.98)** | **0.005** |
| Sex |  |  |
| Male | **ref** |  |
| Female | 0.34 (0.07, 1.57) | 0.17 |
| Race and Ethnicity |  |  |
| White | ref |  |
| Black or African American | 0.28 (0.06, 1.27) | 0.10 |
| Asian | 1.42 (0.32, 6.32) | 0.64 |
| Hispanic or Other Ethnicity** | 2.86 (0.77, 10.54) | 0.12 |
| Insurance Type or Status |  |  |
| Private or Commercial | ref |  |
| Medicaid | 0.59 (0.10, 3.29) | 0.54 |
| Medicare | 0.69 (0.26, 1.84) | 0.46 |
| No Insurance | 1.45 (0.57, 3.66) | 0.43 |
| Patient-Reported Adherence per 1% Increase | 1.00 (0.99, 1.02) | 0.90 |
| **HCQ Dose per 1 mg/kg/day Increase** | **1.45 (1.01, 2.09)** | **0.04** |
| **HCQ Blood Levels per 100 ng/mL Increase** | **0.88 (0.81, 0.95)** | **0.002** |
| Other SLE Medicines |  |  |
| None | ref |  |
| **Disease Modifying Agents (cs-DMARDs), yes** | **5.19 (2.20, 12.25)** | **<0.001** |
| Biologicals, yes | 2.06 (0.64, 6.64) | 0.23 |
| Combination, yes | 1.74 (0.58, 5.30) | 0.32 |
| **Prednisone Dose >5 mg, yes** | **10.44 (3.84, 28.42)** | **<0.001** |
| EGFR per 15 ml/min/1.73m² Increase | 0.75 (0.58, 0.98) | 0.04 |
| SLICC Damage Index (SDI) per 1-Unit Increase | 1.21 (0.92, 1.61) | 0.17 |

Footnotes: Values represent adjusted odds ratios (OR) with 95% confidence intervals (CI), derived from a generalized linear mixed model (GLMM) with random slope and intercept, adjusted for all variables in the model. P values < 0.05 indicate statistical significance. A value < 1.0 indicates lower odds of active systemic lupus erythematosus (SLE) compared with the reference group, while a value > 1.0 indicates higher odds. eGFR = estimated glomerular filtration rate; SLICC = Systemic Lupus International Collaborating Clinics; HCQ = hydroxychloroquine; cs-DMARDs = conventional synthetic disease-modifying antirheumatic drugs. *1 patient with no outcome variable data with 3 visits was excluded. **Includes visits that did not report race or ethnicity.

**Supplementary File 8: Longitudinal Association Between HCQ Whole Blood Levels and SLEDAI-2K Scores Across Visits**

**Supplementary File 9: Generalized Linear Mixed Effects Model Examining the Between-Patient and Within-Patient Effects of HCQ Dose and HCQ Blood Levels on Active SLE (SLEDAI-2K ≥6) Over Time in SLE (n=962*)**

| **Variables at Visit** | **Adjusted Odds Ratio (95% CIs)** | **p-value** |
| --- | --- | --- |
| **Age, per 1-Year Increase** | **0.95 (0.92, 0.99)** | **0.01** |
| Sex |  |  |
| Male | ref |  |
| Female | 0.27 (0.06, 1.21) | 0.09 |
| Race and Ethnicity |  |  |
| White | ref |  |
| Black or African American | 0.44 (0.10, 2.01) | 0.29 |
| Asian | 1.39 (0.34, 5.81) | 0.65 |
| Hispanic or Other Ethnicity** | 2.39 (0.68, 8.44) | 0.17 |
| Insurance Type or Status |  |  |
| Private or Commercial, yes | ref |  |
| Medicaid, yes | 0.37 (0.05, 2.53) | 0.31 |
| Medicare, yes | 0.70 (0.26, 1.89) | 0.48 |
| No Insurance | 1.32 (0.52, 3.36) | 0.56 |
| Patient-Reported Adherence per 1% Increase | 1.00 (0.99, 1.01) | 0.86 |
| HCQ Dose |  |  |
| Within Patients HCQ Dose Increase per 1 mg/kg/day | 1.42 (0.99, 2.02) | 0.054^+^ |
| Between Patients Mean Difference in HCQ Dose in 1 mg/kg/day increments | 1.16 (0.74, 1.83) | 0.52 |
| **HCQ Whole Blood Levels** |  |  |
| **Within Patients HCQ Blood Levels Increase per 100 ng/mL** | **0.67 (0.52, 0.86)** | **0.002** |
| **Between Patients Mean Difference in HCQ Levels in 100 ng/mL Increments** | **0.49 (0.29, 0.83)** | **0.007** |
| Other SLE Medicines |  |  |
| None | ref |  |
| **Disease Modifying Agents (cs-DMARDs), yes** | **3.10 (1.35, 7.15)** | **0.007** |
| Biologicals, yes | 0.90 (0.28, 2.85) | 0.86 |
| Combination, yes | 1.28 (0.44, 3.77) | 0.65 |
| **Prednisone Dose >5 mg, yes** | **9.07 (3.89, 21.17)** | **<0.001** |
| **eGFR per 15 mL/min/1.73m² Increase** | **0.75 (0.59, 0.96)** | **0.02** |
| SLICC Damage Index (SDI) per 1-Unit Increase | 1.16 (0.89, 1.52) | 0.28 |

Footnotes: Values represent adjusted odds ratios (OR) with 95% confidence intervals (CI), derived from a generalized linear mixed model (GLMM) with random slopes and intercepts, adjusted for all variables in the model. P values < 0.05 indicate statistical significance shown in bold font. A value < 1.0 indicates lower odds of active systemic lupus erythematosus (SLE) compared with the reference group, while a value > 1.0 indicates higher odds. + indicates trend towards significance. Abbreviations: eGFR = estimated glomerular filtration rate; HCQ = hydroxychloroquine; cs-DMARDs = conventional synthetic disease-modifying antirheumatic drugs. *1 patient with no outcome variable data with 3 visits was excluded. **Includes visits that did not report race or ethnicity.

| **Supplementary File 10: Generalized Linear Mixed Effects Model Examining the Between-Patient and Within-Patient Effects of HCQ Dose and HCQ Blood Level Categories on Active SLE (SLEDAI-2K ≥6) Over Time in SLE (n=962 visits*)** **Variables At Visit** \| **Adjusted Odds Ratio (95% CIs)** \| **p-value**; **Age, per 1-Year Increase** \| **0.95 (0.92, 0.99)** \| **0.02**; Sex \| \|; Male \| ref \|; Female \| 0.35 (0.07, 1.75) \| 0.20; Race and Ethnicity \| \|; White \| ref \|; Black or African American \| 0.37 (0.09, 1.56) \| 0.17; Asian \| 1.26 (0.29, 5.39) \| 0.76; Hispanic or Other Ethnicity** \| 2.14 (0.59, 7.82) \| 0.25; Insurance Type or Status \| \|; Private Or Commercial \| ref \|; Medicaid \| 0.34 (0.06, 1.98) \| 0.23; Medicare \| 0.60 (0.23, 1.56) \| 0.29; No Insurance \| 1.13 (0.43, 2.93) \| 0.81; PDC per 1% increase \| 1.00 (0.99, 1.01) \| 0.89; **eGFR per 15 mL/Min/1.73m² Increase** \| **0.76 (0.58, 0.98)** \| **0.03**; SLICC Damage Index (SDI) per 1-unit Increase \| 1.12 (0.85, 1.47) \| 0.42; HCQ Dose \| \|; HCQ Dose Change Per 1-Unit Change (Within Patient Effect) \| 1.29 (0.89, 1.87) \| 0.18; Mean HCQ Dose in mg/kg/Day (Between Patient Effect) \| 1.13 (0.74, 1.73) \| 0.56; **HCQ Whole Blood Levels (Categorical)** \| \|; **HCQ Levels <200 (Very Low)** \| **4.76 (1.56, 14.51)** \| **0.006**; HCQ Levels 200-<750 (Sub-Therapeutic Levels) \| 2.04 (0.95, 4.38) \| 0.07^+^; HCQ Levels 750–1149 (Therapeutic Levels) \| ref \|; HCQ Levels ≥1150 (Supratherapeutic Levels) \| 0.68 (0.28, 1.68) \| 0.41; Other SLE Medicines \| \|; None \| ref \|; **Disease Modifying Agents (cs-DMARDs), yes** \| **3.74 (1.66, 8.41)** \| **0.001**; Biologicals, yes \| 1.03 (0.32, 3.31) \| 0.96; Combination, yes \| 1.41 (0.46, 4.34) \| 0.56; **Prednisone Dose >5 mg, yes** \| **9.76 (3.49, 27.31)** \| **<0.001** Footnotes: Values represent adjusted odds ratios (OR) with 95% confidence intervals (CI), derived from a generalized linear mixed model (GLMM) with random slopes and intercepts, adjusted for all variables in the model. P values < 0.05 indicate statistical significance shown in bold font. A value < 1.0 indicates lower odds of active systemic lupus erythematosus (SLE) compared with the reference group, while a value > 1.0 indicates higher odds. + indicates trend towards significance. Abbreviations: eGFR = estimated glomerular filtration rate; SLEDAI = Systemic Lupus Erythematosus Disease Activity Index; HCQ = hydroxychloroquine; cs-DMARDs = conventional synthetic disease-modifying antirheumatic drugs; PDC = Portion of Days Covered. *1 patient with no outcome variable data with 3 visits was excluded. **Includes visits that did not report race or ethnicity. | | | | | | | |
| --- | --- | --- | --- | --- | --- | --- | --- |
| **Supplementary File 11. Summary of Studies Longitudinally Analyzing the Impact of Change in HCQ Blood Levels and SLE Outcomes Over Time (n=7)** | | | | | | | |
| **Author, Year** | **N** | **Time** | **No. of HCQ Levels Per Subject** | **Outcomes** | **Analysis** | **Within/Between Subject** | **Findings** |
| Costedoat-Chalumeau, 2013 | 573 | 7 mos. | 2-6, Levels at 7-month follow-up | SLEDAI-2K | Stepwise multivariate logistic regression analysis | Between | Less flares in patients with higher levels: 13.9% vs 32.7% |
| Cunha et al., 2017 | 171 | 26.9 mos. | 7.5, Mean levels from all visits | Complete/Partial renal remission | Student’s t-test | Between | Low mean HCQ levels in those with LN flares vs. no flares: 590 ng/ml vs. 810 ng/ml |
| Pedrosa et al., 2020 | 82 | 7 mos. | 2, HCQ blood levels at 7-month follow-up categorized as low vs. high | SLEDAI-2K | Multivariate Regression analysis, fisher’s exact test | Between | Flare rate significantly higher with low vs. high levels (<613.5 vs. ≥613.5 ng/mL): 28% vs. 5% |
| Petri et al., 2021 | 739 | 2330 person years | ≥1, Mean levels from all visits | Thrombotic Event | Pooled logistic regression | Between | Every 200 ng/mL increase in HCQ levels reduced thrombosis risk by 13% |
| Zahr et al., 2021  (Childhood-onset SLE) | 55 | 3 years | 3, Individual HCQ levels at each visit | SLEDAI-2K | Non-Linear Mixed-Effect Model | Within and Between | Lower mean HCQ levels in active SLE vs. inactive disease: 536 ±294 vs. 758 ±490 ng/mL |
| Fasano et al., 2023 | 66 | 24 mos. | 2, Mean levels from all visits | SLEDAI-2K | Cox regression | Between | Lower mean HCQ Levels in those with flares vs. no flares: 378.3±185.5 vs. 782.7±646.7 ng/mL |
| Garg et al., 2024 | 42 | 9 mos. | 2, Therapeutic levels vs. subtherapeutic at 6-9-month follow-up | SLEDAI-2K | Multivariable logistic regression | Between | 26% lower odds of flares in those with HCQ blood levels within 750-1200 ng/mL at 9 mos. follow-up |

*Abbreviations: HCQ = Hydroxychloroquine; Mos. = Months; SLEDAI-2K = SLE Disease Activity Index 2000.*
